# Clinical presentation and immunological features of Post-Malaria Neurologic Syndrome: a case report and review of literature

**DOI:** 10.1186/s12936-020-03476-2

**Published:** 2020-11-23

**Authors:** Nadia Castaldo, Carlo Tascini, Paola Della Siega, Maddalena Peghin, Davide Pecori

**Affiliations:** Infectious Diseases Division, Department of Medicine, University of Udine and Azienda Sanitaria Universitaria Integrata Di Udine, 33100 Udine, Italy

**Keywords:** *Plasmodium falciparum*, Malaria, Post-malaria neurological syndrome, Post-infectious encephalitis

## Abstract

**Background:**

Malaria still represents a major health threat, in terms of both morbidity and mortality. Complications of malaria present a diversified clinical spectrum, with neurological involvement leading to the most serious related-conditions. The authors recently encountered a case of a 60-year old Italian man presenting with confusion, language disturbances and Parkinson-like syndrome 3 weeks after complete remission from severe *Plasmodium falciparum* cerebral malaria. Chemical and microbiological analysis revealed aseptic meningitis, diffuse encephalitis and abnormal immune-activation. Re-infection and recrudescence of infection were excluded. Further analysis excluded paraneoplastic and autoimmune causes of encephalitis. A diagnosis of Post-Malaria Neurological Syndrome (PMNS) was finally formulated and successfully treated with high dose of steroids.

**Methods:**

A systematic research of current literature related to PMNS was performed.

**Results:**

151 cases of PMNS were included, the majority of which occurred after severe *P. falciparum* infections. Four main clinical pattern were identified: 37% of the cases presented as “classical” PMNS, 36% presented as delayed cerebellar ataxia (DCA), 18% resembled acute inflammatory demyelinating polyneuropathy (AIDP), and 8% presented as acute disseminated encephalomyelitis (ADEM)-like form. Differentiation between different forms was not always simple, as clinical and radiological findings frequently overlap. Overall, in almost all of the tested cases, cerebrospinal fluid was found pathological; EEG revealed nonspecific encephalopathy in 30% of classical PMNS and 67% ADEM; imaging tests were found abnormal in 92% of ADEM-like forms. Pathogenesis remains unclear. An autoimmune mechanism is the most corroborated pathogenic hypothesis. Overall, the majority of PMNS cases revert without specific treatment. In most severe forms, high dose steroids, intravenous immunoglobulins, and plasmapheresis have been shown to improve symptoms.

**Conclusions:**

PMNS is a disabling complication of malaria. The overall incidence is not known, due to frequent misdiagnosis and under-reporting. Pathogenesis is not also fully understood, but rapid response to immune-modulating treatment along with similarities to auto-immune neurological disease, strongly support a dysregulated immunological genesis of this condition. The lack of randomized controlled studies regarding therapeutic approaches is a major unmet need in this setting. A systematic collection of all the PMNS cases would be desirable, in order to increase awareness of this rare condition and to prospectively investigate the most appropriate management.

## Background

According to the World Health Organization (WHO) report, in 2018 there were 228 million cases of malaria worldwide. Despite a dramatic reduction in mortality by approximately 50% in the last decade, the estimated number of deaths attributed to malaria was 405,000 in 2018, with the highest burden among children living in endemic countries [1].

At the onset of disease malaria can present with multi organ involvement and different clinical spectrums. Nervous system complications can occur during both the acute infection and the convalescence stage, with cerebral malaria (CM) being the most severe form, especially in severe complicated cases. Despite appropriate treatment, permanent and insidious sequelae have been described in 1–3% of the adult cases up to 10% in children. Nonetheless, neurological complications can occur even in non-complicated forms [2–4].

Post-Malaria Neurological Syndrome (PMNS) is a post-infectious complex neurological entity occurring from 2 days up to 2 months after occurrence of malaria. According to clinical and laboratory features, four main patterns have been suggested: (1) Delayed cerebellar ataxia (DCA); (2) Acute inflammatory demyelinating polyneuropathy (AIDP)-like syndrome; (3) Acute disseminated encephalomyelitis (ADEM)-like syndrome; (4) “Classical” post-malaria neurological syndrome (PMNS) [5]. Table 1 provides a detailed description of each form.

**Table 1:** Suggested PMNS main patterns

PMNS pattern	Clinical presentation and definition
Delayed cerebellar ataxia (DCA)	An acute onset self-limiting ataxia, without any other neurological symptoms. It was the first type of post-malaria neurological complication to be described
Acute inflammatory demyelinating polyneuropathy (AIDP)-like, namely Guillain Barré Syndrome	An acute ascending areflexic weakness, with or without sensory impairment. Cranial nerves might be affected, as well (viz. Miller Fisher Syndrome). In our review AIDP-like forms were defined according to Brighton criteria, as the presence of: a) acute onset of bilateral symmetric flaccid paralysis of the limbs and/or of cranial nerve innervated muscles with or without involvement of autonomic system; b) impaired deep tendon reflexes in affected limbs; c) monophasic pattern; d) absence of an alternative diagnosis
Acute disseminated encephalomyelitis (ADEM)-like syndrome	An autoimmune multifocal demyelinating illness. It can be sometimes associated to autoimmune encephalitis and seizures
“Classical” post-malaria neurological syndrome (PMNS)	A self-limiting encephalopathy whose clinical presentation is not included in the previously described syndromes. Classical PMNS scenario may present both motor, sensorial, and psychiatric symptoms

Notwithstanding, the identification of these clinical patterns is not always simple, due to lack of universally accepted definitions, along with a high level of overlap between certain patterns. For example, ADEM-like and PMNS-like patterns share multiple overlapping features, such as multifocal white matter lesions, at times spontaneous recovery, and good response to immune-suppressive therapy. To this extent, imaging is possibly more significant for detecting ADEM-like forms rather than PMNS-like ones [6]. Furthermore, both DCA and AIDP may occur as part of CM as well as in post-malaria illness. The differential diagnosis between PMNS and CM- related illness relies on the time of onset and the presence of parasitaemia [7, 8]. It should be also pointed out that, apart from these main four patterns, PMNS could anecdotally manifest with isolated non-specific neuropsychiatric features, generalized myoclonus and postural tremors [9, 10].

This manuscript focuses on a case of severe PMNS, occurring 20 days after an episode of cerebral malaria due to *Plasmodium falciparum*. A systematic review on PMNS in literature has been conducted as well, as to provide some hints for further studies.

## Case report

On October, 20th, 2019, a 60-year old previously healthy Caucasian man was admitted to the Emergency Department (ED) of University Hospital of Udine (North East Italy) with a comatose condition after 4 days of persistent fever, confusion, and agitation.

He was employed as a road projector in Ouagadougou, Burkina Faso, where he used to spend almost 9 months per year throughout the last 30 years. He reported experiencing many episodes of non-complicated *P. falciparum* malaria throughout his life (malaria diagnosis was established in the local medical centre in Africa by means of Rapid Diagnosic Tests, RDTs), for which he was treated with an artemisinin-based combination therapy. Nevertheless, he always refused to use anti-malarial chemoprophylaxis out of personal choice.

A diagnosis of cerebral *P. falciparum* malaria was formulated in the ED by means of the RDT and blood smear (parasitaemia of 2.5%). No other criteria of cerebral malaria were present.

The patient was subsequently admitted to the Intensive Care Unit, where he underwent orotracheal intubation, life support therapy, and intravenous (IV) treatment with artesunate. Within two days, his neurological condition rapidly improved. *Plasmodium* clearance was documented after 48 h of IV therapy by means of blood smear. On day 3 the treatment was switched to oral dihydroartemisinin-piperaquine for another 3 days. The patient was then discharged on day 7.

On November 10th, (13 days post discharge), the patient woke up from sleep with high fever, intense headache, and acute confusion. His wife reported having noticed abrupt onset of shaking tremors and spatial–temporal disorientation in the morning.

In the ED his body temperature was 39 °C, his blood pressure was in the normal range limits, and his cardiac frequency was 107 beats per minute. He was agitated, and his sensorium went through different stages of consciousness (his Glasgow Coma Scale score went from 9 to 13 in between the phases).

Physical examination revealed normal pupil reactivity. No cranial nerve palsy was noted. His body was shaken by intense postural tremors. Neck stiffness was also present. A more specific neurological examination was difficult to perform due to the state of consciousness. He went from staring and mute, to aggressive behaviour within few minutes. He was severely confused, and exhibited slurred speech.

Biochemical laboratory data showed normal values, as well as the inflammation parameters (including protein C reactive, PCR and procalcitonin-PCT). No signs of meningeal irritation were observed. Recrudescence of malaria was immediately excluded through RDT, linear-amplification mediated PCR (LAMPCR) and blood smear. Cerebrospinal fluid (CSF) analysis at admission revealed a slightly elevated protein level (737 mg/L), and moderate lymphocytic pleocytosis (16 cells/µL, 64% lymphocytes), with normal glucose levels. Oligoclonal bands (OCBs) type 3 were also found positive. CSF autoimmune panel was performed, and no auto-antibody was found.

Bacterial, mycobacterial and fungal examination of both CSF and blood yielded negative results; multiplex polymerase chain reaction (FilmArray Meningitis/Encephalitis panel bioMérieux, Marcy l’Etoile, France) for bacterial, viral and fungal agents causing meningitis and/or encephalitis was negative (Table 2 shows FilmArray panel results). Blood and urine cultures turned out negative. Serological and molecular analysis excluded further major infectious causes of fever and central nervous system infection as well (*Trypanosoma*, *Leptospira*, *Borrelia* spp, *Rickettsia*, *Leishmania*, *Brucella*, *Erhlichia*, *Treponema*).

**Table 2:** Infectious and autoimmune tests on CSF Microbiological data

Direct microscopic analysis	Negative
Culture	Negative
FilmArray Meningitis/Encephalitis (ME) panel (bioMérieux, Marcy l’Etoile, France)	
*Escherichia coli K1* *Haemophilus influenzae* *Listeria monocytogenes* *Neisseria meningitidis* *Streptococcus agalactiae* *Streptococcus pneumoniae* *Citomegalovirus* (CMV) *Enterovirus* *Herpes simplex virus 1* (HSV-1) *Herpes simplex virus 2* (HSV-2) *Human herpes virus 6* (HHV-6) *Human parechovirus* *Varicella zoster virus* (VZV) *Cryptococcus neoformans/gattii*	Negative Negative Negative Negative Negative Negative Negative Negative Negative Negative Negative Negative Negative Negative
Oligoclonal bands	Positive (type 3 pattern)
Autoimmune panel Anti-NMDA R Anti-VGKC complex: spec. LGI1 Anti-VGKC complex: spec. CASPR2 Anti-AMPA 1 R Anti-AMPA 2 R Anti-GABA B R Anti-DPPX R	Negative Negative Negative Negative Negative Negative Negative

Nevertheless, multiple viral serologies yielded positive results, including IgM and IgG for EBV, CMV, HIV, West Nile Virus, Chikungunya, and Thick Born encephalitis. The autoimmune test panel also showed positivity for antinuclear antibodies (ANA) with 1:1280 titre, along with low titres of anti-Jo1, and anti Scl70. These results were interpreted as false positive results due to a probable cross-reaction attributable to polyclonal B lymphocyte activation.

Electroencephalogram (EEG) showed a diffuse slow activity, with no specific pattern. Computerized tomography (CT) scan of the head and magnetic resonance (MRI) were normal. Whole body imaging investigations through CT scan, testicular echography and 18F-fluorodeoxyglucose (18F-FDG) positron emission tomography/computed tomography (PET/CT) were also performed, to exclude any paraneoplastic aetiology.

On day 5, the patient’s neurological condition suddenly declined, with accentuation of four-limb tremors, and occurring of facial and buccal fasciculation, dysgraphia, dysphasia with aggressive and incomprehensible language, hallucinations, and refractory insomnia. Physical examination also revealed bradykinesia, Parkinson-like gait, and postural instability.

CM recurrence or recrudescence was once again excluded by means of RDT, LAMPCR and blood smear. A second lumbar puncture showed a significant increase of CSF cell count (47/µL), with predominance of polymorphonuclear elements (80%), and higher protein concentration (1188 mg/L). Microbiological panel test was confirmed negative.

A diagnosis of PMNS was finally elaborated and IV methylprednisolone was started at the dose of 1 g/day for three days; then the dose was tapered over 4 weeks by oral route. CSF was re-checked on day 10, finding complete normalization of cell count and slight residual hyperproteinorrachia. The neurological condition dramatically improved within 1 day, with fully recovery in 3 weeks.

## Methods

### Data sources

A systematic research of current literature related to PMNS was conducted from January, 1970 to October, 2020. The following keywords and MeSH terms were employed: “Post-malaria neurological syndrome”; post-malaria[All Fields] AND neurological[All Fields] AND ("syndrome"[MeSH Terms] OR "syndrome"[All Fields]);Postmalaria neurological syndrome; Post-malaria ADEM; post[All Fields] AND ("malaria"[MeSH Terms] OR "malaria"[All Fields]) AND ("encephalomyelitis, acute disseminated"[MeSH Terms] OR ("encephalomyelitis"[All Fields] AND "acute"[All Fields] AND "disseminated"[All Fields]) OR "acute disseminated encephalomyelitis"[All Fields] OR "adem"[All Fields]); Post malaria DCA; post[All Fields] AND ("malaria"[MeSH Terms] OR "malaria"[All Fields]) AND DCA[All Fields]; Post-malaria neurological; postmalaria[All Fields] AND neurological[All Fields]; Acute inflammatory demyelinating polineuropathy after malaria, Acute[Title] AND disseminated[Title] AND encephalomyelitis[Title] AND malaria[Title]. The reference databases were: Medline, Pubmed, and Embase. Data from abstracts, poster presentations at congresses, and guidelines were also included. Appendix 1 summarizes the details of Data sources.

### Terms and definitions

*Malaria infection:* the presence of malaria parasites in the blood confirmed by microscopic examination, regardless of the presence or absence of clinical symptoms [1].

*Severe malaria:* a malaria infection presenting with signs and/or symptoms of severity and/or evidence of vital organ dysfunction [1].

*Cerebral malaria:* severe *P. falciparum* malaria occurring with coma (Glasgow coma scale < 11 or Blantyre coma scale < 3) and/or coma persisting for more than 30 min after seizure.

*PMNS:* neurological or psychiatric symptoms occurring in a time-interval from 2 days up to 60 days after a malaria episode, whose parasitaemia have been completely cleared at the time of PMNS occurrence [4].

*PMNS syndromes:* no specific case definition exists for each of the four syndrome. In this review, the cases have been grouped according to current literature regarding both malaria and non-malaria settings. See Table 1 for full definitions.

### Study selection

Overall, 202 records were identified and screened. The publications were evaluated independently by each reviewer, as to exclude duplicated articles, those that were non-pertinent to the review and those not meeting the case definition of PMNS (e.g. neurological entities occurring during acute parasitaemia). Manuscripts in languages other than English, Italian, French, Portuguese, and Spanish were not included as well. Overall, 77 publications were included in the current review.

## Results

This systematic review included 151 cases of PMNS, the majority of which occurred in form of classical PMNS (37%, 56 cases) and DCA (36.4%, 55 cases). AIDP and ADEM represented only a small part of the collected PMNS, respectively 18.5% (28 cases), and 8% (12 cases). Both adult and children were included.

Table 3 summarizes main clinical and diagnostic findings, treatment, and outcomes data.

**Table 3:** Main clinical and diagnostic findings, treatment, and outcomes data

	PMNS	ADEM	AIDP	DCA
Collected cases (n)	56	12	28	55
Demographic informations				
Country of acquisition (n, %)	Africa: 29 (16.2); South and Central America: 2 (3.6); South-West Asia: 23 (41.1); India 1 (1.8)^a^	Africa: 5 (41.6); Australia: 1 (8.3); India: 6 (50)^b^	Africa 11 (29.2); South America 1 (3.6); South West Asia 3 (10.7); India 12 (46.4)^c^	Africa: 1 (1.8); South-West Asia: 42 (76.4); Unknown: 12 (21.8) ^d^
Sex (n, %)	Male: 38 (67.9)	Male: 7 (58.3)	Male: 19 (69.7)	Male: 49 (89.1)
Age (mean, range,, median, IQR range)	34.65,(6–70), 29, (29–50),	29.45, (1–61),29, (11–48)	32.36(6–63), 32.4, (23.2–39.5)	32.3(15–54), 30, (21.5–46.2)
Immune status (n,%)	Naive: 20 (35.7), semi-immune: 7 (12.5), unknown: 29 (51.8)	Naive: 7 (58.3); semiimmune: 5 (41.7)	Semiimmune: 4 (14.3); unknown: 24 (85.7)	Semiimmune: 24 (43.6); unknown: 31 (56.4)
Malaria infection				
Plasmodium species (n,%)	*Falciparum*: 54 (96.4%); *Vivax*: 1 (1.8); mixed *Vivax* and *Falciparum*: 1 (1.8);	*Falciparum*: 6 (50%); *Vivax*: 4 (33.3%); mixed *Vivax* and *Falciparum*: 1 (8.3)	*Falciparum*: 22 (78.6); *Vivax*: 5 (17.9), Mixed: none	*Falciparum*: 54 (98.2); *Vivax*: 1 (1.8), Mixed: none
Severe forms (n,%)	44 (78.6)	8 (66.7)	1 (3.6), unknown 20 (71.4)	55 (100)
Post-malaria neurological syndrome: clinic and diagnostic tests				
Presentation (n,%)	Neuropsychiatric disturbances: 14 (25), in 1 (1.8) not reported; seizures: 39 (69.6); motor disturbances (including tremors and cerebellar syndrome): 38 (67.9), in 1 (1.8) not reported	Neuropsychiatric disturbances: 6 (50); seizures: 5 (41.7); motor disturbances (including tremors and cerebellar syndrome): 12 (100)	Neuropsychiatric disturbances: 0, in 22 (78.6) not reported; seizures: 0, not reported in 22 (78.6); motor disturbances (including tremors and cerebellar syndrome): 28 (100)	Neuropsychiatric disturbances: 0; seizures: 0, motor disturbances (including tremors and cerebellar syndrome): 55 (100)
Pathological MRI (n,%)	8 (14.3); in 30 (53.6) not reported	11 (91.7)	1 (3.6); in 27 (96.4) not reported	0; in 54 (98.2) not reported
Pathological EEG (n,%)	16 (28.6); in 37 (66.0) not reported	8 (66.7%)	In 28 (100%) not reported	0; 43 (78.2) not reported
Pathological CSF (n; %)**	44 (84.6); in 4 (7.1) not reported	10 (100); in 2 (16.6) not reported	15 (88.2); in 11 (39.3) not reported	0; 55 (98.8) not reported
CSF cells/uL*	25.15 (± 43.55),predominantly lymphocytes	29,45 (± 19,96), predominantly lymphocytes	0.94 (± 1.43)	1, predominantly lymphocytes
CSF proteins mg/L	1164.2 (± 3039.16)	1563.00 (± 2080.9)	1444.12 (935.6)	400
Post-malaria neurological syndrome: treatment				
Supportive measures alone (n,%)	41 (73.2)	1 (8.3)	13 (72.2); in 10 not reported	2 (66.7), in 51 not reported
OTI need (n,%)	4 (7.1)	1 (8.3)	0	0
Steroids (n,%)	15 (26.8)	11 (91.7)	2 (11.1); in 10 not reported	2 (66.7); in 51 (92.7) not reported
IV immunoglobulin (n,%)	0	0	2 (11.1); in 10 not reported	0; in 51 (92.7) not reported
Plasma exchange (n,%)	0	0	1 (5.3); in 9 not reported	0; in 51 (92.7) not reported
Outcome				
Death (n,%)	1 (1.8)	0	7 (25)	3 (5.5)
Sequelae (n,%)	0	1 (8.3)	20 (71.4)	43 (78.2)
Complete recovery (n,%)	55 (98.2)	11 (91.7)	1 (3.6)	9 (16.4)
Peculiarities	Multiple blood serology positivity (IgM and IgG): 5 (8.9) Oligoclonal IgM bands on CSF: 1 (1.8) Autoimmune antibodies positivity on blood: 4 (7.14) Pathological PET-TC: 1 (1.8) Pathological SPECT: 1 (1.8) Pathological EMG/ENG: 1 (1.8) Autonomic system disorder: 1 (1.8)	Autoimmune antibodies positivity on blood: 2 (16.6) MRI lesions mimicking MS: 1 (8.3) Pathological VEP: 1 (8.3)	Pathological EMG: 8 (28,6) Monolateral 7th nerve palsy: 1 (3.6)	Autoimmune antibodies positivity on blood: 1 (1.8)

*PMNS* post-malaria neurological syndrome; ADEM: acute disseminated encephalomyelitis,* AIDP* acute inflammatory demyelinating polyneuropathy,* DCA* delayed cerebellar ataxia,* MRI* Magnetic Resonance Imaging,* EEG* electroencephalogram,* CSF* cerebrospinal fluid,* OTI* Orotracheal Intubation,* IV* intravenous,* VEP* Visual evoked potentials,* PET/TC* Positron Emission Tomography/Computed Tomography,* SPECT* Single Photon Emission Computed Tomography,* EMG/ENG* eletromyo/neurography

^a^Africa 1 (1.8); Benin, Togo, Burkina 1 (1,8); Brazil 1 (1.8); Congo 1 (1.8); Dominican Republic 1 (1.8); Ghana 1 (1.8); Guinea 1 (1.8); Guinea and Congo 1 (1.8); India 1 (1.8); Kenya 1 (1.8); Madagascar 1 (1.8); Malawi 1 (1.8); Mali and Benin 1 (1.8), Mali 2 (3.6), Mozambique 1 (1.8); Nigeria 1 (1.8); Sierra Leone 1 (1.8), Tanzania 1 (1.8); Togo 1 (1.8); West Africa 1 (1.8); Cameroon 2 (3.6); Gambia 3 (5.4); Angola 4 (7,1); Ivory Coast 3 (5.0); Sri Lanka and Thailandia 23 (41.1)

^b^Angola1(8.3), Ivory Coast 1 (8.3), Nigeria 1 (8.3), Sierra leone 1 (8.3), Vanatu 1 (8.3), West Africa 1 (8.3), India 6 (50)

^c^Brazil1 (3.6); Malawi 1 (3.6), Nepal 1 (3.6), Sri Lanka 1 (3.6), Thailand 1 (3.6), Sudan 10 (25.7), India 13 (46.4)

^d^Ghana1 (1.8); Sri Lanka 42 (76.4); Unknown 12 (21.8)

**CSF is defined pathological when any one of the following is present: increased cells (≥5/µL), increased protein concentration (≥450 mg/L), low glucose concentration (≤50 mg/dL and/or 2/3 of blood glucose concentration)

*No data about LCR in 5 patients (8.9) in PMNS, 2 (16.7) in ADEM, 13 (46.4) in AIDP, and 54 (98.2) in DCA

Overall, more than half of the patients were males, with mean ages of 35 years in classical PMNS, 29 years in ADEM, and 32 years in AIDP and DCA. Immune status profiles of the patients varied widely. In classical PMNS, 35.7% (20 patients) were naïve, and 12.5% (7 patients) were semi-immune. In ADEM 58.3% of the cases (7 patients), were naive. Immune status was not specified in most of AIDP and DCA cases.

Classical PMNS occurred following *P. falciparum* infection in 96.4% of the cases (54 patients); the majority of these infections had been acquired in South-West Asia (23 cases, 41.1%) and Africa (29 cases, 16.2%). ADEM mainly occurred following *P. falciparum* infections (6 cases, 50%) and *Plasmodium vivax* infections (4 cases, 33.3%). The countries were occurred most frequently were India (6 cases, 50%), and Africa (5 cases, 41.6%). AIDP was associated with *P. falciparum* infection in 78.6% of the cases (22 patients). *Plasmodium vivax* infections occurred in 17.9% (5 patients) of AIDP. As for ADEM, most of the malaria infections preceding AIDP were acquired in India (12 cases, 46.4%), and Africa (11 cases, 29.2%). DCA generally followed *P. falciparum* infections (98.2%, 54 cases out 55), and were mainly localized in South-West Asia (76.4%, 42 cases). Mixed infections (*P. vivax* and *P. falciparum*) were reported in a few cases.

Malaria infection presented as complicated form in 78.6% (44 cases) of classical PMNS, 66.7% (8 cases) of ADEM, and 100% (55 cases) of DCA. Severe malaria was reported in only one patient (3.6%) presenting with AIDP. Noteworthy, characteristics of malaria infection were not clarified for most of AIDP cases.

Clinical manifestations varied widely. Neuropsychiatric disturbances were the most frequent symptoms in classical PMNS (25%, 14 cases) and ADEM (6 cases, 50%), whereas they were never reported in either AIDP or DCA. Similarly, seizures occurred in almost 70% (39 cases) of classical PMNS, and 41.7% (5 cases) of ADEM. No seizures were reported in AIDP and DCA. Various degrees of motor disturbances were described in most forms (including tremors, cranial nerve palsy, pyramidal, and cerebellar syndrome). Motor disorders occurred in 67.9% (38 cases) of classical PMNS and in 100% of ADEM, AIDP and DCA.

Instrumental tests showed a low diagnostic yield in the majority of the reports. Head magnetic resonance (MR) showed non-specific alterations in white matter in 14.3% (8 cases) of classical PMNS and 91.7% (11 cases) in ADEM. In most of AIDP and DCA reports, instrumental tests were not described. EEG generally showed a diffuse encephalopathy with generalized slow pattern. Pathological EEG patterns were described in 28.6% (16 cases) of classical PMNS and 66.7% (8 cases) of ADEM. Again, for AIDP and DCA there is a lack of information regarding EEG results.

Overall, lumbar puncture was performed and described in 53.6% of the cases (81 patients). Abnormal physical–chemical composition of CSF was detected in 84.6% of the “classical PMNS” (44 out of 52 patients), 88.2% of AIDP (11 out 17 patients) and 100% of the ADEM (10 cases out 10). An elevated cell count was observed in most cases (predominantly lymphocytes) with or without elevated protein concentration levels. In 1 classical PMNS case, intrathecal production of IgM was reported [5]. Within the DCA group, CSF analysis was performed in one case [11]; no abnormal values were reported.

With regards to treatment, the most frequent approach was symptomatic. Overall, general supportive measures without any specific therapy (such as antipyretics, oxygen therapy and anti-epileptic drugs) were attempted in 73.2% of the classical PMNS (41 cases), 46.4% of ADEM (13 cases), 72.2% of AIDP (13 cases), and 66.7% of DCA (2 cases). Among the patients who received specific treatments besides supportive measures, corticosteroids were administered in 92% of ADEM (11 patients), 27% of classical PMNS (15 cases), 11% of AIDP (2 cases) and 66.7% (2 patients) of DCA. Furthermore, in AIDP intravenous immunoglobulins and plasma exchange methods were administered and employed in 11.1% (2 cases) and 5.3% (1 case), respectively.

Fatal outcome was reported in 1.8% of classical PMNS (1 case), 5.5% of SCA (3 cases), and 25% (7 cases) of AIDP. No deaths were reported in the ADEM group. Almost all the survivors fully recovered from classical PMNS and ADEM. Various grade of neurocognitive impairment and motor sequelae was described in 71.4% of AIDP (20 cases) and 78.2 (48 cases) of DCA.

## Discussion

PMNS has been defined as a complex neurological condition developing within 2 months after fully recovery from a malaria episode [4]. The first reports date back to 1966. Since then a number of case reports and case series of PMNS have been described. Incidence rates of PMNS has been estimated to range from 1.2 per 1000 cases in uncomplicated forms, up to 18 out of 1000 in severe forms. PMNS after *P. falciparum* infections are the most frequently reported [4]. Noteworthy, most of the PMNS presenting as ADEM occurred after non-falciparum malaria [6], but the reason for this association is not clear.

### Clinical presentation

Clinical findings in “classical PMNS” are extremely heterogeneous, ranging from acute encephalitis in almost 80% of the patients, confusion and fever in over half of the cases, seizures (33%), language disturbance (about 30%), tremor (23%), myoclonus (11%), and psychiatric illness (17%) [5]. Neurocognitive decline, acalculia, and, rarely, autonomic system disorders, ophtalmoplegia and cranial nerve palsy have been described as well. DCA is most typically characterized by gait ataxia and isolated cerebellar syndrome, whereas in ADEM-like forms motor impairment in predominant [8, 12].

### Diagnostic methods

Overall, instrumental tests seemingly did not represent valuable diagnostic tools in any of the described cases. In a minority of reports, MR showed nonspecific signal uptake in white matter. The most frequent abnormalities are localized in periventricular areas, basal ganglia, brain stem, and cerebellum. Spinal cord and optic nerve involvement may also occur albeit more rarely [13]. Commonly, the diagnosis of ADEM is suggested by MRI multifocal, bilateral, asymmetric, white matter abnormalities, with hyperintense appearance in T2 and FLAIR sequences [14]. Localization of MR lesions is thus unrelated to clinical presentation (e.g., ataxia does not necessarily correlates with cerebellar lesions) [8].

### Pathogenesis

An immune-mediated process is the most corroborated hypothesis regarding PMNS pathogenesis. Different mechanisms have been proposed.

Auto-reacting T-cells could be triggered by a certain grade of molecular mimicry and nonspecific activation, leading to autoimmune response toward CNS antigens [6, 15]. This mechanism is also a well-known prerequisite of classical AIE, AIDP and ADEM.

In the wake of the immunologic-theory, same authors described a series of PMNS cases developing as classical autoimmune encephalitis (AIE) mediated by neuronal antibodies against ions channels and synapses. Alike AIE, PMNS has been associated to the production of N-methyld-aspartate-receptor -NMDAR, antibodies, anti-voltage-gated-potassium-channel (VGKC) antibodies, anti neuroanexin α3 antibodies. In these cases, the disappearance of autoantibodies and MRI lesions have been documented following steroid treatment cycles [16, 17].

Polyclonal B cell over-activation is another possible immunological mechanism. It has been demonstrated that *Plasmodium*-parasitized erythrocytes express several membrane microbial immunoglobulin binding proteins (IBPs), which persist over time following parasite eradication. Some IBPs, such as *P. falciparum* erythrocyte membrane protein 1 (PfEMP1) extensively bound to different circulating human immunoglobulins, thus leading to direct B-lymphocyte stimulation and subsequent secretion of different antibodies [18]. This theory might explain the frequent finding of elevated titres of IgG and IgM antibodies against multiple viruses during PMNS. Despite the exclusion of any concomitant infectious disease as underlying cause of the encephalopathy, the patient presented various degrees of IgG and IgM positivity against multiple viruses, low positivity for ANA, CSF lymphocytic pleocytosis and intrathecal immunoglobulin production.

The case study protagonist developed a severe form of PMNS characterized by a dramatic neuropsychiatric pattern with abnormal generalized immune-activation and severe blood brain barrier alteration. Indeed, clinical presentation, immunological findings, and rapid response to steroids endorsed the immune-mediated trigger theory.

Two alternative hypotheses regarding PMNS pathogenesis have been postulated so far: 1) transient ischaemia; 2) cytokine storm.

The ischaemic hypothesis suggests that parasitized red blood cells adhere to endothelia and reversibly obstruct the brain microvasculature. It has been proposed that the higher tendency to cytoadhere of *P. falciparum* might explain the higher prevalence of PMNS in this species [19]. Hsieh et al*.* reported a case of PMNS where brain SPECT revealed decreased radiolabelled agent incorporation in cerebral hemispheres, hence suggesting impairment of cerebral microcirculation [20, 21]. However, the absence of parasitaemia during PMNS and the time laps between malaria episodes and post-infectious syndrome remains unexplained. Furthermore, an ischaemic genesis is much more compatible with cerebral malaria than PMNS.

The cytokine storm hypothesis derived from a single in vivo study. de Silva HJ et al*.* conducted a prospective observational analysis in 12 patients with post-malaria DCA, reporting significantly higher levels of pro-inflammatory cytokines, such as tumour necrosis factor (TNF), interleukin 6 (IL-6) and interleukin 2 (IL-2), in both serum and CSF, and comparing PMNS affected patients to non PMNS affected ones (8 patients). Indeed, TNF levels in CSF have been linked to more severe and disabling forms of CM [22, 23]. It has been suggested that TNF might play a pathogenic role in CNS damage in CM, by possibly promoting parasite sequestration and endothelial activation. A similar role may be hypothesized also in PMNS. However, no prospective analysis has been conducted to date in post-malarial disorders.

### The role of anti-malarial treatment

During the past decade, a series of studies suggested that the type of anti-malarial treatment could correlate to occurrence of PMNS. In particular, the administration of mefloquine and atovaquone-proguanil was recognized as a risk factor for PMNS [24, 25]. The quinoline anti-malarials (and especially mefloquine) have been associated to neurological disorders, both in form of psychiatric symptoms, and central anticholinergic syndrome [4]. Some of these manifestations overlap with PMNS.

However, to date no significant association of PMNS with anti-malarial treatment has been found.

Firstly, quinoline-related effects are self-limiting, and they rapidly fade. None of the anti-malarial drugs has been related to iatrogenic ADEM-like, AIP, or DCA-like toxicity. Furthermore, not all the patients presenting with PMNS received quinolines. Notably, most of the cases emerged days or weeks after quinoline-withdrawal.

### Treatment and outcome

Overall, the majority of PMNS cases revert without specific treatment. Prognosis is generally good and no long-term sequelae have been described. Several off-label treatments have been proposed for most severe forms. High dose steroids have been administered in the majority of the reported cases. Overall, in this revision 30 patients (19.8%) received steroids, the majority of which in the classical PMNS group (15 cases) and the ADEM-like group (11 cases). The most frequently applied therapeutic schedule consisted in IV administration of either 1 g/day of methylprednisolone or equivalent dosage of prednisolone administered over 5–7 doses, and subsequent tapering over 4–6 weeks. Alternative dosages included: IV methylprednisolone at 100 mg/day for three doses, and tapered for 10 days; oral prednisolone at 60 mg/day for 4 days without tapering [26, 27]. The first schedule was chosen.

In almost all the reported cases, a rapid improvement of symptoms was observed upon steroids administration. Similarly, the condition of the patient improved rapidly after the first dose of corticosteroid and his neurological impairment fully reverted after 3 weeks. It has been suggested that steroids might hasten the resolution of PMNS through anti-inflammatory effect and immune-suppression, as to turn-off the auto-immune trigger. Notably, steroids have been associated to sensible reduction of serum and CSF concentrations of inflammatory cytokines [24]. Nonetheless, the impact of steroid treatment on outcome and sequelae has not been analysed in any of the current studies. To date, there is no evidence that steroids either ameliorates the prognosis, reduces the sequelae, or affect mortality rates in PMNS. Nevertheless, corticosteroids might be harmful in patients suffering of cerebral malaria, as they might increase the risk of seizures and gastrointestinal haemorrhages [28]. It is not clear whether steroids really changes the course of the disease, as some of these forms might have probably spontaneously revert without treatment.

**Fig. 1:**
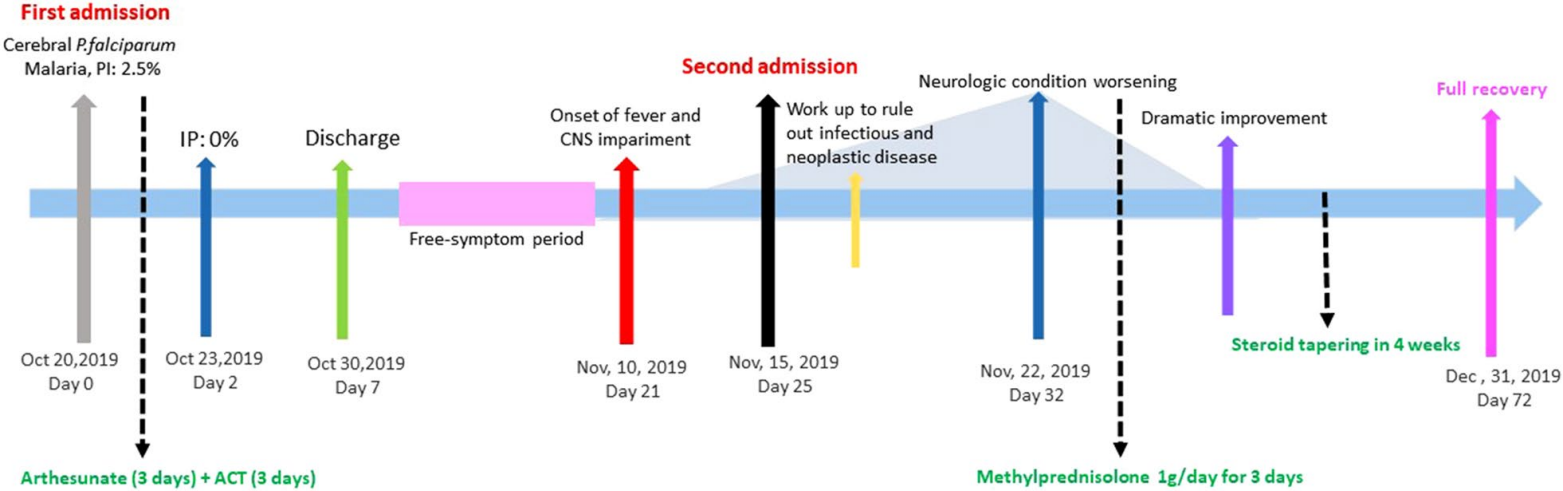
summarizes clinical case-timeline

Some authors promote the use of intravenous immunoglobulins (IV-Ig) in refractory cases as a second line therapy. Indeed, IV-Ig have been demonstrated to exert beneficial effects by inhibiting and reverting the cytoadherence of infected erythrocytes in vitro [29]. Stangel et al*.* were the first group to report the ability of polyclonal immunoglobulins in vitro to modulate nitric oxygen production and microglial function in vitro. It has been assumed that IV-Ig modulate the local immune response also in CNS [30]. Further modes of action of IV-Ig have been attributed to the ability of hyperimmune Ig clones to bind and neutralize circulating antibodies and activated B cells, as well as to modulate auto-immune response [31, 32]. Marchioni et al*.* described a case series of 5 patients affected by steroid-resistant post-infectious (non post-malaria) ADEM and myelitis which successfully reverted upon IV-Ig [33]. A two patient-case series by Ravaglia et al. described more extensively the IV-Ig role on specific functional systems and long-term sequelae, also suggesting a potential synergy between IV-Ig and steroids [34]. However, both the aforementioned case series included patients affected by post-infectious neurological complications, but none of them specifically included cases of PMNS (Fig. 1).

Plasmapheresis is another salvage therapy, which could be considered in most severe PMNS, in analogy with post-infectious encephalitis [26]. In this review, plasma exchange was successfully performed in one case of severe AIDP after *P. falciparum* malaria [35, 36].

In summary, there is lack of consensus regarding PMNS management. There is currently no available data comparing the “watchful waiting strategy”” with the immunomodulatory treatments. The actual efficacy of steroids, IV-Ig, and plasmapheresis is currently under debate.

A course of steroids should be attempted in severe non-self limiting forms (e.g. those with no improvement or progressive worsening in few days after the presentation). Second-line treatment with IV-Ig should be considered for steroid-refractory disease, especially as regards “classical” PMNS. A combination of steroids and IV-Ig is strongly recommended especially in those patients presenting with ADEM-like forms.

To date plasmapheresis has been considered a rescue strategy for progressive non-resolving disease. However, it could be attempted as a first-line treatment for AIDP-like forms.

## Conclusion

PMNS is a serious complication of malaria. The precise epidemiology is yet unclear, as diagnosis is often subject to misinterpretation due to heterogeneous clinical presentation and diagnostic features are heterogeneous. Pathogenesis is not also fully understood, but rapid response to immune-modulating treatment along with similarities to auto-immune neurological disease, strongly support a dysregulated immunological genesis of this condition.

Aetiologic treatment is not available, but corticosteroids, IV-Ig administration, and plasmapheresis may offer certain benefits in the most severe cases. Clinicians should be encouraged to consider PMNS in patients presenting with neurological symptoms after a malaria episodes. A systematic collection of all the PMNS cases is thus desirable, in order to increase awareness of this rare condition and to prospectively investigate the most appropriate management.

## Figures and Tables

**Fig. 2:**
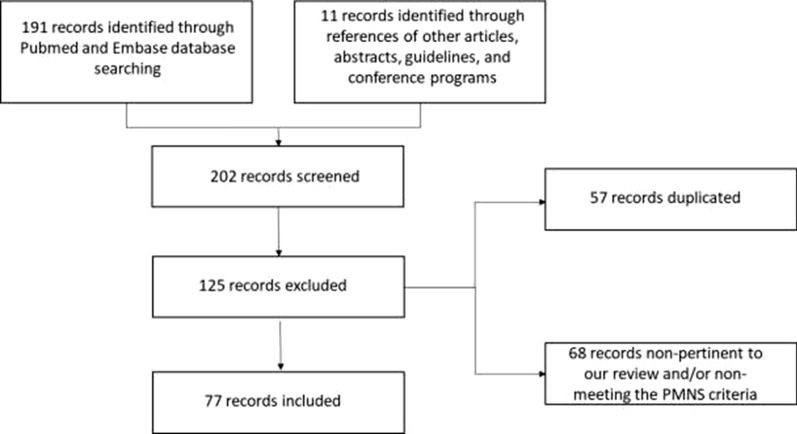
Study selection process

## Data Availability

Available upon request.

## Appendix

See Fig. 2.
